# The volume of the thalamus and hippocampus in a right-handed female episodic migraine group

**DOI:** 10.3389/fneur.2023.1254628

**Published:** 2023-10-19

**Authors:** Mingchen He, Gréta Kis-Jakab, Hedvig Komáromy, Gábor Perlaki, Gergely Orsi, Edit Bosnyák, Renáta Rozgonyi, Flóra John, Anita Trauninger, Kata Eklics, Zoltán Pfund

**Affiliations:** ^1^Pécs Diagnostic Center, Pécs, Hungary; ^2^The Hungarian Research Network-Pécsi Tudományegyetem, Clinical Neuroscience Magnetic Resonance Research Group, Pécs, Hungary; ^3^Department of Neurosurgery, Medical School, University of Pécs, Pécs, Hungary; ^4^Department of Neurology, Medical School, University of Pécs, Pécs, Hungary; ^5^Department of Languages for Biomedical Purposes and Communication, University of Pécs, Pécs, Hungary

**Keywords:** episodic migraine, white matter lesions, migraine headache characteristics, volume of thalamus and hippocampus, right-handed female migraineurs

## Abstract

**Background/aim:**

Migraine is a disabling headache with clinical and radiological complications. The aim of this study was to investigate the volume of the thalamus and hippocampus in migraineurs, the role of white matter lesions (WMLs), and the migraine characteristics in volume changes.

**Methods:**

Brain MRIs of 161 right-handed female episodic migraine patients and 40 right-handed, age-related, healthy women were performed. Left and right thalamus segmentation was performed on the 3D MPRAGE images using the Freesurfer 5.3 image analysis suite. Hippocampal subfield segmentation was based on a novel statistical atlas built primarily upon ultra-high-resolution *ex vivo* MRI data.

**Results:**

The left hippocampus had a smaller and the left thalamus had a larger total volume than the right one in both the control (*p* < 0.001) and migraine groups (*p* <0.001). Patients with white matter lesions (L+) showed smaller right thalamus and right hippocampal tail volumes than patients without lesions (L–) (*p* = 0.002 and *p* = 0.015, respectively) and controls (*p* = 0.039 and *p* = 0.025, respectively). For the right hippocampal body, we found significantly smaller volume in L+ patients when compared to L– patients (*p* = 0.018) and a similar trend when compared to the control group (*p* = 0.064). Patients without aura (A–) showed a larger right hippocampus (*p* = 0.029), right hippocampal body (*p* = 0.012), and tail volumes (*p* = 0.011) than patients with aura (A+). Inverse correlations were found between attack frequency and the volumes of the left and right hippocampal tails (*p* = 0.018 and *p* = 0.008, respectively).

**Conclusion:**

These findings indicate that WMLs may influence the volume of the right thalamus and hippocampus, while migraine aura and attack frequency may lead to volume changes in different parts of the hippocampi in migraine patients. These data support the necessity of effective migraine management to limit subcortical volume loss in migraineurs.

## Introduction

Migraine is a painful, returning headache disease that lasts for decades (1). Migraine is more prevalent in women than men due to hormonal differences starting in puberty (2–4). In addition, the migraine characteristics of men and women are different. Women report a longer attack duration, increased risk of headache recurrence, higher intensity of headaches, more frequent nausea, phonophobia, and photophobia, greater disability, and a longer time required for recovery (2–4).

Magnetic resonance imaging (MRI) is a useful diagnostic tool in migraine and shows migraine-related structural complications, e.g., hemispheric white matter lesions (WMLs) as well as cortical and subcortical volume changes of different structures (5, 6). The WMLs are clinically mute, mostly progressive microvascular tissue damages, which may influence the intrahemispheric size and volume impairment (5). Previously, we studied the possible influence of WMLs on hemispheric cortical thickness and volume in 161 right-handed female migraineurs, but statistically significant correlations were not found (6).

In healthy adults, the total brain volume was reported to be larger from birth in men than in women by ~11%. This size difference accounts for other reproducible findings: higher white/gray matter ratio, intra- vs. interhemispheric connectivity, and regional cortical and subcortical volumes in men. When structural and lateralization differences are present independent of size, sex/gender explains only ~1% of the total variance (7). Furthermore, there are several documented structural and functional brain differences between men and women, but it is hard to decide which of them is sex hormone-related (8).

The trigeminovascular system is a pain-transmission link between the vascular (dural and cortical) and neuronal (brainstem and thalamus) regions (9). The thalamus is a relay station; thereby, it has high (density) connectivity to many brain structures. It holds an important position in allodynia, central sensitization, and photophobia in migraine (10).

The hippocampus is involved in memory consolidation, spatial navigation, and pain-related stress response (11), as well as in pain processing, pain-related attention, and anxiety (12). Stronger hippocampal-cortico-limbic connectivity in migraineurs is associated with allodynia (11).

Based on the current literature, both structures are functionally impaired in migraine; thereby, we hypothesized that the hemispheric tissue damage (WMLs) and the migraine characteristics (disease duration, frequency, and aura) may negatively affect the volume of both the thalamus and hippocampus.

To test our hypothesis, we reinvestigated our abovementioned migraine headache patients' brain MRI studies (6) to measure the volume of the thalami and hippocampi and to evaluate the potential role of WMLs and the migraine characteristics in volume abnormalities.

## Patients and methods

### Subjects

A total of 161 women with episodic migraine (mean age 39.3 ± 12.5, range 18–73 years; disease duration 15.6 ± 11.9, range 1–57 years; attack frequency/month 5.6 ± 4.5, range 0.2–20.0; 63 with aura; 52 with white matter lesions; Table 1) were prospectively enrolled in this study. As controls, 40 age-matched healthy female subjects were included (mean age 38.3 ± 10.0, range 19–66 years). Migraineurs had no other types of headaches. None of the included migraine patients' headaches or auras were unilaterally side-locked in nature, but the headaches showed right hemisphere dominance in 68.3% (right-sided or bilateral with right > left pain intensity). An MRI was performed during a headache-free period for each patient; the minimum headache-free period was 1 day after the postdrome headache phase. All migraineurs and controls reported right-handedness. All subjects were strictly investigated for medical comorbidities (e.g., past and current medical history, physical examination, routine and autoimmune blood tests, cardiovascular monitoring, BMI, sleep disease, hypertension, gynecological disease, thyroid gland, and cognitive disease), and only those were enrolled in this study who lacked any medical diseases. Brain MRI studies of healthy participants did not show any structural abnormalities.

**Table 1:** Demographic and clinical data of migraine patients and healthy controls.

	**Lesion+ (*n* = 52)**	**Lesion- (*n* = 109)**	**Controls (*n* = 40)**	**Differences (** ***p-*** **value)**		
				**L+ vs. L–**	**L+ vs. C**	**L– vs. C**
Age (years)	44.6 ± 13.1 (20–72)	36.7 ± 11.4 (18–73)	38.3 ± 10.0 (19–66)	< 0.001^a^	0.018^a^	0.422^a^
Disease duration (years)	19.8 ± 12.9 (1–57)	13.7 ± 10.9 (1–43)	–	0.003^a^	–	–
Migraine attack frequency/month	5.4 ± 4.2 (0.2–17.0)	5.7 ± 4.6 (0.5–20.0)	–	0.807^a^	–	–
Patients with aura	31 (59.6 %)	32 (29.4 %)	–	< 0.001^b^	–	–

Lesion+, migraineurs with lesions; Lesion–, migraineurs without lesions. Values are given as mean ± standard deviation (minimum–maximum).

^a^Mann-Whitney *U*-test (two-sided exact *p*-value).

^b^Fisher's exact test (two-sided exact *p*-value).

### MRI acquisition

All subjects were scanned on the same 3T MRI scanner (Magnetom TIM Trio, Siemens AG, Erlangen, Germany) using a 12-channel head matrix coil. A whole-brain T1-weighted three-dimensional axial magnetization-prepared rapid gradient-echo (3D MPRAGE) sequence was acquired using the following parameters: TR/TI/TE = 1,900/900/3.4 ms, BW = 179 Hz/px, flip angle = 9°, FOV = 210 × 240 mm^2^, matrix size = 224 × 256, slice thickness = 0.94 mm, 176 axial slices, 0.94 × 0.94 × 0.94 mm^3^ isotropic voxels. Beyond the routine T1- and T2-weighted measurements, the scanning protocol also included fluid-attenuated inversion recovery (FLAIR) imaging (TR/TI/TE = 13,200/2,600/100 ms, BW = 401 Hz/px; echo trains = 14, FOV = 186 × 220 mm^2^, matrix size = 162 × 192, slice thickness = 1.5 mm, 100 axial slices).

### MR image analysis

Left and right thalamus segmentation was performed on the high-resolution three-dimensional 3D MPRAGE images using the Freesurfer 5.3 image analysis suite (https://surfer.nmr.mgh.harvard.edu/fswiki). The technical details were described previously (13). Quality control was performed throughout the automatic processing stream. When the reconstruction was inaccurate, error correction was performed based on the recommended workflow (http://surfer.nmr.mgh.harvard.edu/fswiki/RecommendedReconstruction). The left and right hippocampus and their subfields (head, body, and tail) were segmented using the development version of FreeSurfer on 31 August 2017 (https://surfer.nmr.mgh.harvard.edu/fswiki/DownloadAndInstall). This new hippocampal subfield segmentation method is based on a novel statistical atlas built primarily upon ultra-high-resolution (~0.1 mm isotropic) *ex vivo* MRI data. This *ex vivo* atlas outperforms the *in vivo* counterpart that was distributed with Freesurfer 5.3 and yields subregion volumes that agree better with those from histological studies. The technical details of this new method were described earlier (14).

### Analysis of white matter lesions

**Figure 1:**
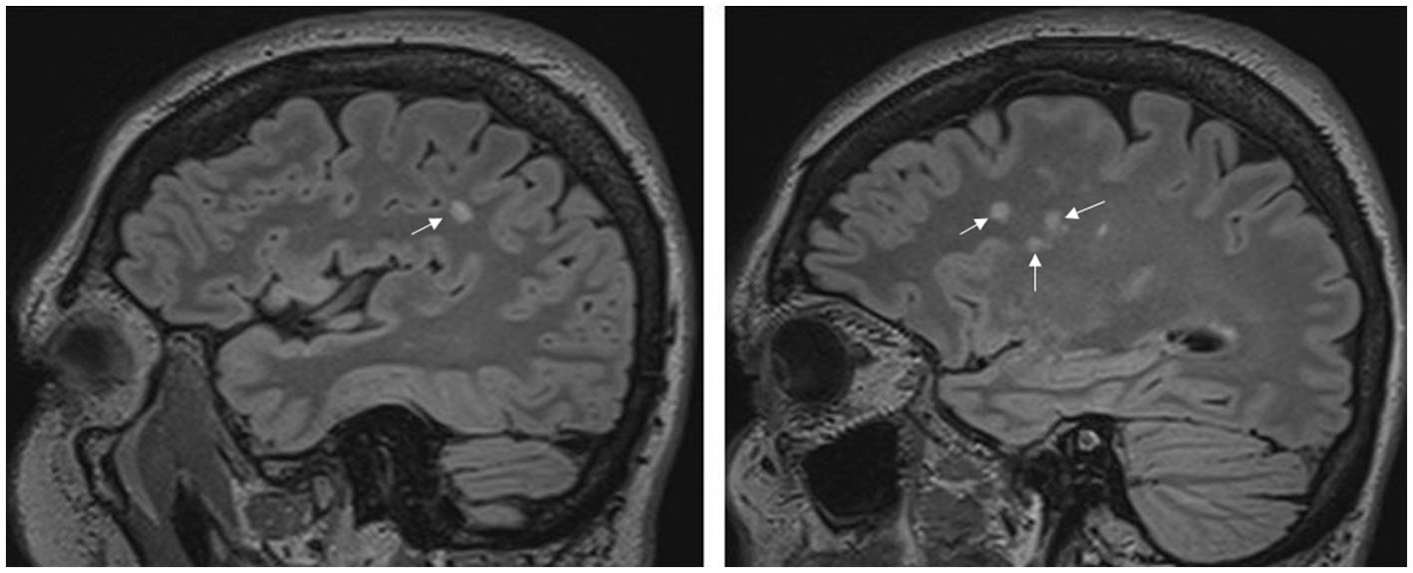
Migraine patient with intrahemispheric white matter lesions. The sagittal fluid-attenuated inversion recovery (FLAIR) images belong to one patient and show high signal intensities in the white matter. The arrows show the lesions.

**Figure 2:**
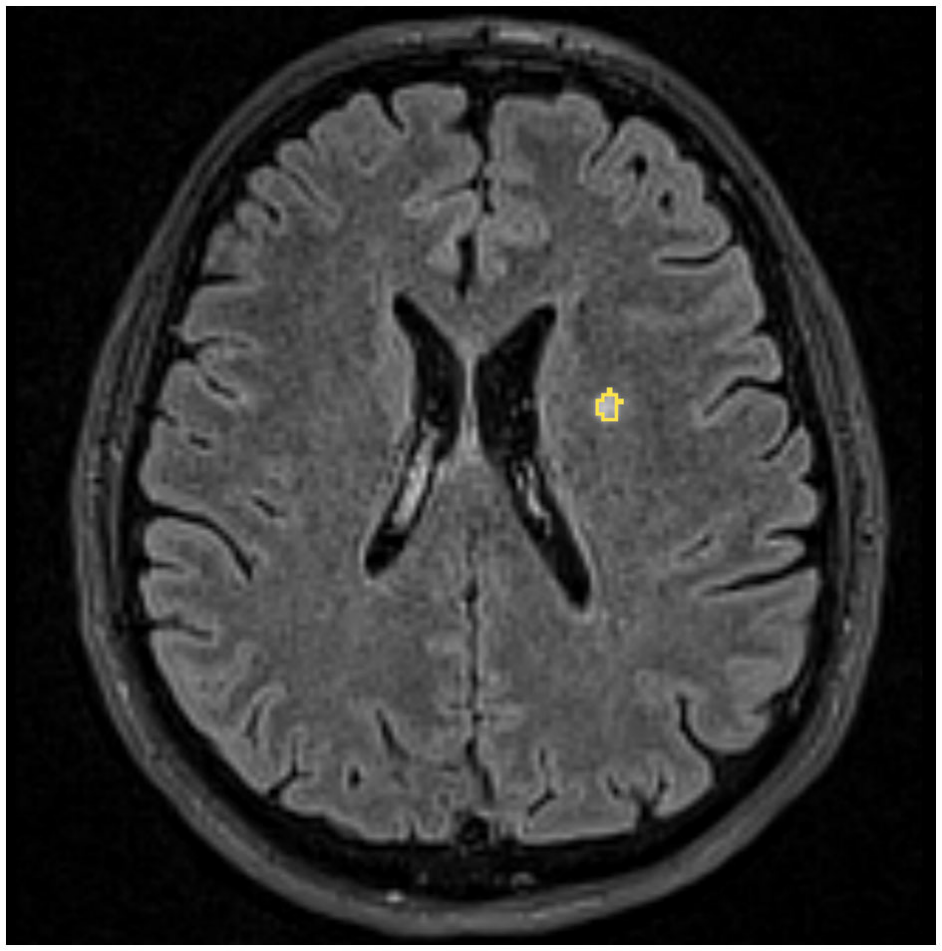
Delineation of a white matter lesion in a female migraineur. An example of an axial FLAIR image used to assess white matter lesion is shown in the background. Yellow indicates the outline of a manually delineated white matter lesion.

WMLs were considered if visible as hyperintensity on T2-weighted and FLAIR MRI but without a low-signal intensity lesion on T1-weighted MRI and larger than 3 mm, appearing in at least two consecutive slices (Figure 1). Supratentorial WMLs were delineated manually on the FLAIR images using the 3D Slicer software (http://www.slicer.org, Version 4.6.2, Figure 2). Total and lobar hemispheric WML numbers/volumes were calculated for all L+ subjects. The borders of the lobes were defined as previously described (6).

### Statistical analysis

Statistical analyses were performed using the SPSS 20.0 software (IBM Corp., Armonk, NY, United States).

Differences in age between the whole migraine group and healthy controls were assessed using the Mann-Whitney *U*-test. Age differences among migraine subgroups (L+ and L–) and the healthy control group were assessed using the Kruskal–Wallis test followed by the Mann-Whitney *U*-test. The age difference between migraine patients without aura (A–) and those with aura (A+) was assessed using the Mann-Whitney *U*-test.

A Wilcoxon signed-rank test was used to identify whether there were statistically significant differences between the total volume of the left and right hippocampus, as well as between the left and right thalamus, separately in the healthy control group and the migraine group. Volume differences between the healthy control group and the migraine group in the left and right hippocampus, as well as the thalamus, were assessed by ANCOVA using age and total intracranial volume (ICV) as covariates.

Differences in continuous migraine-related variables (i.e., disease duration, migraine attack frequency) between migraine subgroups (L+ and L–) were compared using the Mann–Whitney *U*-test, while differences in the rate of aura (A+ or A–) between the same subgroups were assessed using Fisher's exact test. Within the migraine group with lesions (L+), a Wilcoxon signed-rank test was used to test for differences between left and right hemispheres in total and lobar hemispheric WML numbers/volumes. These differences were not tested for the temporal and occipital lobes because lesions were rare in these lobes, with a median lesion number of 0.

Differences in the thalamus, hippocampal, and hippocampal subfield volumes among the three groups (L+, L–, and control) were assessed using ANCOVA with age and ICV as covariates. In the case of significance, the least significant difference *post-hoc* analyses were conducted for pairwise comparison of the three groups.

Within the whole migraine group, the same volumes were compared between patients with aura (A+) and without aura (A–) using ANCOVA with age and ICV as covariates. The possible effects of other migraine characteristics (i.e., disease duration and migraine attack frequency) on the examined volumes were tested using stepwise multiple linear regression analyses. In these models, age, ICV, lesion, and aura group variables were also included as possible predictors.

Within the migraine group with lesions (L+), we assessed the potential relationships between left and right hemispheric brain structure volumes and the total and lobar lesion numbers/volumes in the corresponding hemisphere. Since the lesion number/volume data were not normally distributed (right-skewed with several extreme values), to perform powerful statistical analyses, the effects of lesion numbers/volumes were assessed by creating binarized subgroup variables based on the median split of them, e.g., the subgroup with a low number/volume (below or equal to the median) vs. the subgroup with a high number/volume of left hemispheric frontal lobe lesions. The effects of these subgroup variables on the examined volumes were tested using stepwise multiple linear regression analyses, including all these subgroup variables, age, and ICV as possible predictors. Since lesions were rare in the temporal and occipital lobes with a median lesion number of 0, the effects of lesion number/volume in these lobes were not assessed. Results were considered significant at *p* ≤ 0.05.

## Results

There was no significant difference in age between the whole migraine group and the control group (*p* = 0.736). The Kruskal–Wallis test revealed significant age differences among the migraine subgroups (L+ and L–) and controls (*p* = 0.001). *Post-hoc* testing indicated that the L+ subgroup was significantly older than the other two groups (Table 1). There was no significant difference in age between patients without aura (A–) and with aura (A+) (*p* = 0.816, two-sided exact *p*-value).

The Wilcoxon signed-rank test showed that the left hippocampus had a smaller total volume when compared with the right hippocampus in both the healthy control group (3,353 ± 252 mm^3^ vs. 3,517 ± 289 mm^3^, *Z* = −4.968, *p* < 0.001) and the migraine group (3,379 ± 293 mm^3^ vs. 3,494 ± 317 mm^3^, *Z* = −8.653, *p* < 0.001). On the other hand, the left thalamus had a bigger volume when compared with the right thalamus in both the healthy control group (7,708 ± 747 mm^3^ vs. 6,984 ± 562 mm^3^; *Z* = −5.275, *p* < 0.001) and the migraine group (7,675 ± 817 mm^3^ vs. 6,951 ± 689 mm^3^, *Z* = −10.835, *p* < 0.001). There were no significant differences between the healthy control group and the whole migraine group in the total volume of the left hippocampus (*p* = 0.703), right hippocampus (*p* = 0.436; one outlier patient was excluded based on standardized residuals > 3), left thalamus (*p* = 0.801; one outlier patient was excluded based on standardized residuals < −3), and right thalamus (*p* = 0.726).

Disease duration and the rate of aura were significantly higher in L+ patients, while migraine attack frequency was not significantly different between the L+ and L– subgroups (Table 1). Results for the number and volumes of supratentorial white matter lesions in L+ patients in different hemispheric lobes and the total left and right hemispheres are presented in Table 2. The Wilcoxon signed-rank test indicated that there were no differences between the left and right hemispheres in total (*p* = 0.341), frontal (*p* = 0.084), and parietal (*p* = 0.264) WML numbers, and no differences were found for the total (*p* = 0.448) and frontal (*p* = 0.226) lesion volumes. However, the lesion volume was bigger in the left compared to the right-hemispheric parietal lobe (*Z* = −2.097, *p* = 0.036).

**Table 2:** Number and volumes of supratentorial white matter lesions in the Lesion+ migraine subgroup.

**Location**	**Left hemisphere**		**Right hemisphere**	
	**Lesion number**	**Lesion volume (mm^3^)**	**Lesion number**	**Lesion volume (mm^3^)**
Total	5 (0–41)	171 (0–4,343)	5 (0–39)	153 (0–4,632)
	8	355	8.75	354
Frontal lobe	3 (0–24)	68 (0–1,542)	3 (0–26)	92 (0–3,283)
	5	231	6	180
Parietal lobe	1 (0–11)	31 (0–2,558)	0.5 (0–11)	6 (0–1,749)
	4	167	3	115
Temporal lobe	0 (0–4)	0 (0–274)	0 (0–6)	0 (0–658)
	1	27	1	21
Occipital lobe	0 (0–2)	0 (0–183)	0 (0–2)	0 (0–374)
	0	0	0	0

Lesion+, migraine patients with lesions. Values are given as median (minimum–maximum) and interquartile range, separately for each hemispheric lobe and for the total left and right hemispheres.

After correcting for the confounding effects of age and ICV, the patients with lesions (L+) showed significantly smaller right thalamus and right hippocampal tail volumes than patients without lesions (L–) and controls (Table 3). For the right hippocampal body, the ANCOVA showed a strong trend (*p* = 0.051); therefore, we performed the *post-hoc* tests, which indicated a significantly smaller volume in L+ patients when compared to L– patients and a trend when compared to the control group (Table 3).

**Table 3:** Group differences in the volumes of examined structures among lesion-related migraine subgroups and controls.

	**Groups**			**Statistics**				
	**L+**	**L–**	**Control**	**ANCOVA test**		**LSD** ***post-hoc*** **test**		
				***F* (df1, df2)**	***P***	**L+ vs. L–**	**L+ vs. C**	**L– vs. C**
L thalamus	7,548 (914)	7,758 (727)	7,708 (747)	1.639 (2,194)	0.197	–	–	–
R thalamus	6,698 (689)	7,072 (659)	6,984 (562)	**5.092 (2,195)**	**0.007**	**0.002**	**0.039**	0.560
L hippocampus	3,340 (345)	3,397 (265)	3,353 (252)	0.735 (2,195)	0.481	–	–	–
R hippocampus	3,434 (364)	3,522 (289)	3,517 (289)	1.722 (2,195)	0.181	–	–	–
L hippocampal head	1,689 (194)	1,709 (157)	1,685 (124)	0.311 (2,195)	0.733	–	–	–
R hippocampal head	1,742 (215)	1,756 (156)	1,762 (160)	0.288 (2,194)	0.750	–	–	–
				0.438^a^ (2,192)	0.646^a^			
L hippocampal body	1,150 (102)	1,158 (84)	1,140 (80)	0.903 (2,189)	0.407	–	–	–
R hippocampal body	1,153 (115)	1,191 (94)	1,185 (103)	3.022 (2,194)	0.051	**0.018**	0.064	0.928
L hippocampal tail	504 (70)	524 (62)	518 (67)	1.672 (2,194)	0.191	–	–	–
				1.776^b^ (2,193)	0.172^b^			
R hippocampal tail	539 (71)	567 (67)	570 (56)	**3.589 (2,195)**	**0.029**	**0.015**	**0.025**	0.766

L+, migraine patients with lesions; L–, migraine patients without lesions; ICV, total intracranial volume; L, left; R, right; LSD, least significant difference.

Subjects were excluded from the analysis if the corresponding standardized residuals from the ANCOVA model were below −3 or above 3. A maximum of three subjects had to be excluded from either group. Volumes are presented as uncorrected mean (standard deviation) in mm^3^ for subjects included in the ANCOVA statistics. For *post-hoc* tests, *P*-values are given.

^a^Statistical values are also presented for the group effect at mean ICV when including the significant Group^*^ICV interaction term in the model.

^b^Statistical values are also presented for the group effect when including the significant Age^*^ICV interaction term in the model.

Bold values denote statistical significance.

Focusing on the migraine group, patients without aura (A–) showed a larger right hippocampus, right hippocampal body, and tail than patients with aura (A+) (Table 4).

**Table 4:** Group differences in the volumes of examined structures between aura-related migraine subgroups.

	**Groups**		**ANCOVA test**	
	**Aura+**	**Aura-**	***F* (df1, df2)**	***P***
L thalamus	7,655 (942)	7,688 (729)	0.295 (1,157)	0.588
R thalamus	6,966 (765)	6,941 (640)	0.003 (1,157)	0.955
L hippocampus	3,339 (328)	3,404 (267)	3.004 (1,157)	0.085
R hippocampus	3,439 (346)	3,529 (293)	**4.849 (1,157)**	**0.029**
L hippocampal head	1,685 (192)	1,714 (153)	1.947 (1,157)	0.165
R hippocampal head	1,739 (208)	1,759 (154)	1.115 (1,156)	0.293
			1.141^a^ (1,155)	0.287^a^
L hippocampal body	1,143 (106)	1,164 (96)	2.615 (1,156)	0.108
R hippocampal body	1,158 (107)	1,192 (98)	**6.504 (1,156)**	**0.012**
L hippocampal tail	512 (67)	524 (66)	1.654 (1,157)	0.200
R hippocampal tail	542 (63)	569 (71)	**6.669 (1,157)**	**0.011**
			**6.998**^b^ **(1,156)**	**0.009** ^b^

Aura+, migraine patients with aura; Aura-, migraine patients without aura; ICV, total intracranial volume; L, left; R, right.

Subjects were excluded from the analysis if the corresponding standardized residuals from the ANCOVA model were below −3 or above 3.

Maximum one subject had to be excluded from the Aura- group. Volumes are presented as uncorrected mean (standard deviation) in mm^3^ for subjects included in the ANCOVA statistics.

^a^Statistical values are also presented for the group effect when including the significant Age^*^ICV interaction term in the model.

^b^Statistical values are also presented for the group effect at mean ICV when including the significant Group^*^ICV interaction term in the model.

Bold values denote statistical significance.

**Figure 3:**
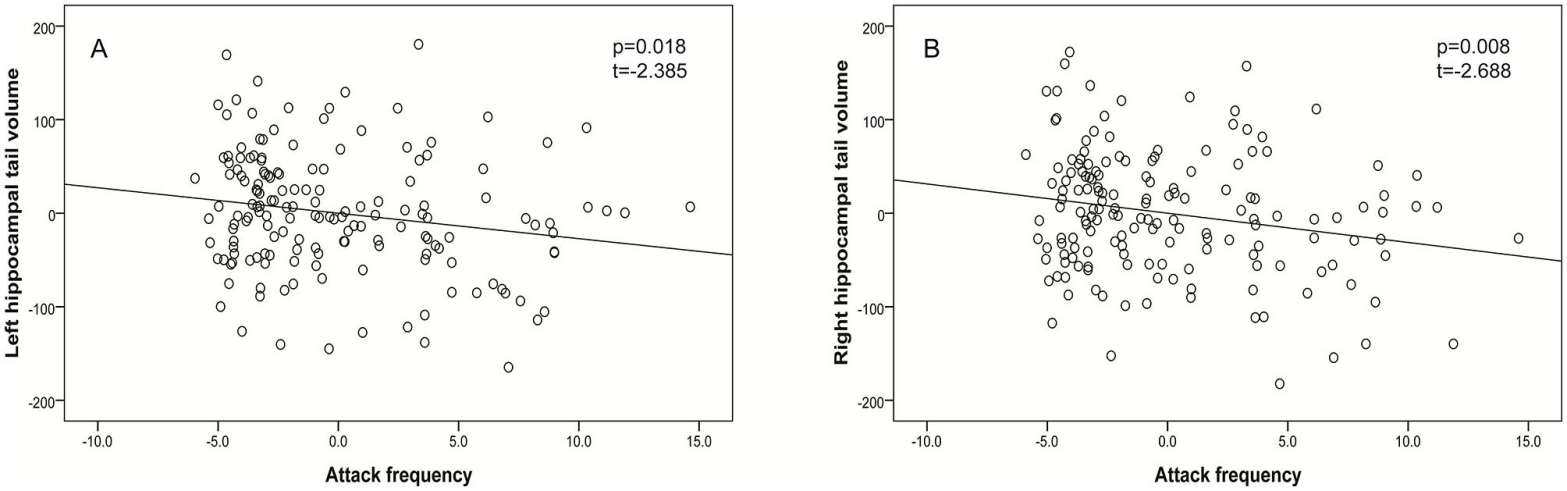
Partial regression plots demonstrating that more frequent migraine attacks were associated with decreased volumes of the left hippocampal tail (after controlling for the effects of age and ICV, **A**) and the right hippocampal tail (after controlling for the effects of age, ICV, lesion, and aura, **B**).

Within the whole migraine group, neither the disease duration nor migraine attack frequency was selected by stepwise linear regression as significant predictors of the examined structural volumes, except for the volumes of hippocampal tails. Using multiple linear regression, including the volume of the left hippocampal tail as the dependent variable and migraine attack frequency, age, and ICV as independent variables, we found a significant negative correlation between attack frequency and the volume of the left hippocampal tail (*p* = 0.018, *t* = −2.385, Figure 3A). Based on our results reported in Tables 3, 4, for the right hippocampal tail, we also included the lesion (L– vs. L+) and aura (A– vs. A+) in addition to migraine attack frequency, age, and ICV as independent variables in the multiple linear regression model. We found a negative correlation of attack frequency with the right hippocampal tail volume (*p* = 0.008, *t* = −2.688, Figure 3B).

In the L+ migraine group, none of the binarized total or lobar hemispheric lesion number/volume variables were selected by stepwise linear regression as significant predictors of the examined structural volumes.

## Discussion

In this rather homogenous patient population, we investigated 161 right-handed episodic migraineurs. To eliminate the confounding effect of sex-related differences in headache characteristics and brain size, only women were enrolled in the study. The total volumes of the left or right thalami and hippocampi were not different between migraineurs and controls. The volume of the right thalamus was smaller than the left one within the migraine and control groups. Patients with WMLs showed a smaller right thalamus than in lesion-negative migraineurs. The hippocampi were smaller on the left side in both groups. The hippocampus subgroup analysis of migraine patients and the subfield volume values of all three groups showed statistically significant differences. The white matter lesions, the presence of aura, and the attack frequency appeared to be risk factors for smaller volumes. It is also possible that the dominant unilateral headache side with more attacks on the right side has a role in the volume differences of the thalami. Since the patients' memory was only reliable for the last month, we did not use these data for statistical analysis.

### Thalamic and hippocampal volume asymmetries in healthy subjects

In our right-handed healthy female control group, significant volume differences were detected between the thalami (R < L) and hippocampi (R > L) of the two hemispheric sides. Since in the cited morphometric studies (15–17), the participants were not separated by sex, and handedness was not documented in one study, these findings are just partly comparable with our data. However, handedness may influence the hippocampus volume on the same side.

### Manual dominance in migraine

In a large retrospective study, the authors collected data from 546 patients with migraine aged between 16 and 65 years, reporting manual dominance according to the Edinburgh test. They included 466 right-handed and 80 left-handed subjects with migraine and registered 4,215 unilateral painful attacks. It was concluded that manual dominance influences the side of pain lateralization in migraine (18). Considering our 1-month-long database, the right-handed data are congruent.

### The volume of the thalamus in migraine

In a study of 35 patients with migraine without aura and 40 healthy controls, the authors found thalamic nuclei with significantly increased and decreased volumes. The authors concluded that the result might contribute to the underlying pathogenesis of migraine (19).

In another study, significant volume reductions of three thalamic nuclei were observed in migraineurs: the central nuclear complex, the anterior nucleus, and the lateral dorsal nucleus (20). The thalamic nuclei with reduced volumes were connected to the limbic system.

In measurements of thalamic subregions in 77 migraineurs with episodic migraine and 30 controls, selective functional hypoconnectivity in the thalamic subregions was detected (21). The data provided neuroimaging evidence of thalamocortical pathway dysfunction in episodic migraines, including emotion and personality traits in migraineurs.

In a recent thalamic study, the volume was investigated by analyzing MRI data obtained from a large study, which specifically included women migraineurs with aura, unrelated migraine-free matched controls, and migraine aura-free co-twins. None of the analyses showed any between-group differences in the volume of the thalamus or of individual thalamic nuclei. These results indicated that the pathophysiology of migraine with aura did not involve alteration of thalamic volumes (22). According to our findings, thalamic volume abnormalities were not detected in migraine aura patients in the latter publication, but we found WML-associated smaller volume.

### The volume of the hippocampus in migraine

In migraine patients, after a long follow-up for 2 years, the right hippocampus volume was positively associated with a good migraine outcome after adjustment of headache frequency (OR 4.72, *p* = 0.024) (23).

In a review of hippocampal volume, a longitudinal study discovered decreased volume in newly diagnosed migraine patients after 1 year (24). A cross-sectional study also suggested an adaptive increase in volume at low headache frequency and a maladaptive decrease in volume at higher headache frequency (25). These results were interpreted as either initial adaptive plasticity of the hippocampus or a larger hippocampus (i.e., a pre-existing condition) in migraineurs that then decreases with increased attack frequency (26). Evidence for the first interpretation has come from animal models of neurogenesis in the structure that is known to persist into adulthood. Stress (e.g., migraine) may mediate adaptive structural plasticity through the remodeling of dendrites and synapses (27). With repeated stress involved in migraine attacks, including pain, glucocorticoids, cortical spreading depression, and gonadal hormones, elevated and prolonged levels of excitatory amino acids are likely released (28, 29). Moreover, the aging rat hippocampus displays elevated and prolonged levels of excitatory amino acids released during acute stress. It is probable that structural plasticity in response to repeated stress starts as an adaptive and protective response but ends up as damage if the imbalance in the regulation of the key mediators is not resolved. It is likely that morphological rearrangements in the hippocampus impair memory functions, and it is conceivable that these may also have a role in chronic pain perception (30). In another study, the authors concluded that migraineurs had smaller hippocampal volume and stronger hippocampal-cortico-limbic connectivity compared to healthy subjects. Hippocampal volumes and measures of hippocampal volume connectivity with other cortico-limbic network regions are associated with symptoms of allodynia (11). These studies show hippocampal volume changes and support the role of migraine frequency in volume abnormalities. Since we also found a hippocampal tail volume association with migraine frequency, these findings are consistent with our results.

### WMLs and migraine characteristics

The thalamic and hippocampal volume changes are likely the consequence of recurrent migraine headaches and accumulate with migraine years (5, 31). The WMLs are worsening with longer migraine disease duration, but they can be found at any age of migraineurs (5). A previous MRI study of supratentorial white matter hyperintensities in migraine patients demonstrated tissue damage with axonal loss, decreased glial cell density with impaired energy metabolism, enlarged extracellular space with an increased extracellular water fraction, and decreased blood flow and volume (31). In addition, reactive oxygen species (ROS) are the contributors to oxidative stress, which can cause vascular endothelial dysfunction in migraines and may lead to tissue damage in WMLs (32).

Both the thalamus and hippocampus are involved in migraine bout-related pain processing, which thereby causes stress. These include headache intensity, frequency, and the dominant hemisphere attack. The pain may have a role in the volume changes, especially in the hippocampi.

Migraine aura is usually unilateral and visual, but it can also be sensory, motor, brainstem, and retinal or cause speech/language disturbance. The cortical spreading depression (CSD) is a slowly propagated wave of depolarization of neurons and glial cells, followed by a subsequent sustained suppression of spontaneous neuronal activity, accompanied by complex and variable changes in vascular caliber, blood flow, and energy metabolism. Although spreading depression (SD) has been most extensively studied in the cortex, the phenomenon may occur in all neural tissues, including the hippocampus, cerebellum, and retina, among other regions (33, 34). Growing evidence suggests that an increased propensity to CSD could be a mechanism involved in the increased prevalence of migraine in women. Both estrogen and progesterone have been reported to increase the frequency of CSD (35).

Beyond the careful new migraine patient selection, the short- or long-term follow-up studies give hope for longitudinal remeasurements of the same patient group, to see further volume changes.

## Limitations

The main limitation of this study is that migraine patients were not followed for at least 1 year to obtain precise data on the pain side. Another limitation is the cross-sectional nature of the study, which does not allow for an evaluation of whether the observed volume changes are progressive during the disease course.

We did not adjust *p*-values for multiple comparisons. However, to avoid data dredging, our investigation was restricted to a small number of brain regions predefined based on *a priori* hypothesis. The choice of thalamus and hippocampus was based on previous studies, and we have good reasons to believe that both could be involved in migraine. Adjusting *p*-values for multiple comparisons is still controversial and not always the right choice, especially in studies with a clearly defined primary hypothesis (36).

Thalamus segmentation was performed by Freesurfer 5.3. Having analyzed quite a few of our initial measurements with this version, we did not want to switch to another major version released in the meantime. Such an update is discouraged without repeating all the analyses already completed by Freesurfer 5.3.

## Conclusion

The major findings of this study are the following: (1) the volume of the right thalamus is decreased in the white matter lesion migraine subgroup, (2) the volume of the right hippocampus is smaller in migraineurs with aura and WMLs, and (3) the volume of both hippocampus tails is smaller in patients with a higher migraine frequency. These structural abnormalities are likely to be the consequence of recurrent migraine headaches and their negative impact on the traditional migraine characteristics and progressive nature of WMLs. These processes may negatively influence the function of the thalamus and hippocampus in the human brain. These data further support the importance of effective migraine management; briefly, the patients need to follow a healthy lifestyle and avoid almost all migraine risk factors, and the doctors need to investigate co-morbidities if suspected and control the attacks with the most effective therapy.

## Data availability statement

The raw data supporting the conclusions of this article will be made available by the authors, without undue reservation.

## Ethics statement

The studies involving humans were approved by Regional Research Ethics Committee of the Medical Center, Pécs. The studies were conducted in accordance with the local legislation and institutional requirements. The participants provided their written informed consent to participate in this study.

## Author contributions

MH: Conceptualization, Formal analysis, Methodology, Writing—review and editing. GK-J: Data curation, Investigation, Writing—review and editing. HK: Formal analysis, Writing—review and editing. GP: Conceptualization, Data curation, Formal analysis, Investigation, Methodology, Writing—original draft, Writing—review and editing. GO: Formal analysis, Writing—review and editing. EB: Data curation, Investigation, Writing—review and editing. RR: Data curation, Investigation, Writing—review and editing. FJ: Data curation, Investigation, Writing—review and editing. AT: Data curation, Investigation, Writing—review and editing. KE: Writing—original draft, Writing—review and editing. ZP: Conceptualization, Data curation, Investigation, Methodology, Writing—original draft, Writing—review and editing.
